# MiR-100 overexpression attenuates high fat diet induced weight gain, liver steatosis, hypertriglyceridemia and development of metabolic syndrome in mice

**DOI:** 10.1186/s10020-021-00364-6

**Published:** 2021-09-06

**Authors:** Christian Smolka, Delia Schlösser, Catherine Hohnloser, Xavier Bemtgen, Caterina Jänich, Laura Schneider, Julien Martin, Dietmar Pfeifer, Martin Moser, Peter Hasselblatt, Christoph Bode, Sebastian Grundmann, Franziska Pankratz

**Affiliations:** 1 Department of Cardiology and Angiology I, University Heart Center Freiburg - Bad Krozingen, Medical Center - University of Freiburg, Faculty of Medicine, University of Freiburg, Freiburg, Germany; 2 Department of Hematology, Oncology and Stem Cell Transplantation, Medical Center - University of Freiburg, Faculty of Medicine, University of Freiburg, Freiburg, Germany; 3 Department of Medicine II, Gastroenterology, Hepatology, Endocrinology, and Infectious Diseases, Medical Center - University of Freiburg, Faculty of Medicine, University of Freiburg, Freiburg, Germany

**Keywords:** miR-100, CD36, Fatty acid uptake, Liver steatosis, Metabolic syndrome

## Abstract

**Background:**

Diet-induced obesity can result in the development of a diverse spectrum of cardiovascular and metabolic diseases, including type 2 diabetes, dyslipidemia, non-alcoholic liver steatosis and atherosclerotic disease. MicroRNAs have been described to be important regulators of metabolism and disease development.

**Methods:**

In the current study, we investigated the effects of ubiquitous miR-100 overexpression on weight gain and the metabolic phenotype in a newly generated transgenic mouse strain under normal chow and high fat diet and used microarray expression analysis to identify new potential target genes of miR-100.

**Results:**

While transgenic overexpression of miR-100 did not significantly affect weight and metabolism under a normal diet, miR-100 overexpressing mice showed a reduced weight gain under a high fat diet compared to wildtype mice, despite an equal calorie intake. This was accompanied by less visceral and subcutaneous fat development and lover serum LDL cholesterol. In addition, transgenic miR-100 mice were more glucose tolerant and insulin sensitive and demonstrated increased energy expenditure under high fat diet feeding. A comprehensive gene expression profiling revealed the differential expression of several genes involved in lipid storage- and metabolism, among them CD36 and Cyp4A14. Our data showed a direct regulation of CD36 by miR-100, leading to a reduced fatty acid uptake in primary hepatocytes overexpressing miR-100 and the downregulation of several downstream mediators of lipid metabolism such as ACC1, FABP4, FAS and PPARγ in the liver.

**Conclusions:**

Our findings demonstrate a protective role of miR-100 in high fat diet induced metabolic syndrome and liver steatosis, partially mediated by the direct repression of CD36 and attenuation of hepatic lipid storage, implicating miR-100 as a possible therapeutic target in liver steatosis.

**Supplementary Information:**

The online version contains supplementary material available at 10.1186/s10020-021-00364-6.

## Background

Metabolism defines the entirety of finely regulated biochemical processes which convert fuel from food into energy to facilitate the organism’s growth, survival and development. To maintain this energetic balance, cells have to be constantly provided with energy and substrates necessary to synthesize nucleic acids, carbohydrates, proteins and lipids. This is enabled by a complex orchestra of metabolic pathways as well as nutrient sensors in tissues and organs. Hyperalimentation can misbalance this metabolic equilibrium, resulting in obesity, dyslipidemia and insulin resistance and a severely increased risk for secondary cardiovascular and hepatic diseases (Almeida et al. 2021; Kiran et al. 2021; Mikusova et al. 2021; Rivera-Gonzalez et al. 2021; Singh et al. 2021; Vural Keskinler et al. 2021; Yao et al. 2021).

MicroRNAs (miRNAs) are short, non-coding RNAs which mostly regulate gene expression by interaction with the 3′UTR of their target mRNAs. MiRNAs play a pivotal role as fine tuners of metabolic processes in mammals (Rottiers and Naar 2012) and several “metabomiRs” have been shown to regulate cholesterol and fatty acid metabolism as well as to influence non-alcoholic liver steatosis and insulin resistance (Gerin et al. 2010; Horie et al. 2010; Li et al. 2020; Marquart et al. 2010; Najafi-Shoushtari et al. 2010), thereby adding a new complexity to the metabolic network.

Additionally, many miRNAs have been shown to be differentially regulated in white adipose tissue of obese patients compared to non-obese individuals (Arner and Kulyte 2015; Martinelli et al. 2010; Ortega et al. 2010). Heneghan et al. showed that the expression of miR-17-5p and miR-132 differed significantly between obese and non-obese omental fat and the expression of these two miRNAs in blood and omental fat significantly correlated with leptin, body mass index (BMI) and fasting blood glucose (Heneghan et al. 2011). Keller et al. found an increased expression of miR-21 in human obesity accompanied with a positive correlation of BMI. Many other studies suggest a modification of miRNA expression in mouse models of obesity (Iacomino and Siani 2017), e.g. miR-342-3p, miR-142-3p, miR-142-5p, miR-21 and miR-379 have shown to be upregulated and miR-122, miR-133b and miR-1 to be downregulated during the development of obesity in mice (Chartoumpekis et al. 2012). These findings highlight the role of miRNAs in the field of obesity and related diseases, suggesting the modulation of miRNA expression as potential therapeutic strategy in diseases related to metabolic syndrome.

Our own group recently, identified a protective role of miR-100 in chronic vascular inflammation by direct suppression of mTOR, finally resulting in reduced expression of endothelial adhesion molecules. Surprisingly, we also found enhanced serum triglyceride and cholesterol level under pharmacological inhibition of miR-100 in mice fed with high fat diet (HFD), indicating an influence of this miRNA on metabolic processes (Pankratz et al. 2018).

Here, we describe an exclusive transgenic mouse strain globally overexpressing miR-100. Starting with a comprehensive gene expression profile in liver tissue we identified two genes downregulated by miR-100 namely Cyp4A14 and CD36, which are known to be involved in lipid metabolism and storage. Although we observed no changes neither in the genotype nor phenotype of our transgenic mice under normal diet, adding the metabolic stressor HFD revealed an altered lipogenic gene profile and fat composition in miR-100 overexpressing mice, overall pointing to a beneficial effect of miR-100 in HFD induced liver steatosis.

## Material and methods

### Animals

For all experiments, only male mice were used to minimize data variation which might occur due to the estrous cycle of female animals. C57BL/6J animals were purchased from Charles River or from local stock of the animal facility at the University Hospital Freiburg, Germany. The ubiquitous miR-100 overexpressing mice were obtained by breeding our transgenic BLU.miR-100loxp mice with the CMV-Cre deleter strain (B6.C-Tg(CMV-cre)1Cgn/J, The Jackson Laboratory). All mice were kept under a 12 h/12 h light/dark cycle in a specific pathogen-free animal facility with normal chow diet (ND) and water available ad libitum. For the induction of metabolic stress, resulting in obesity, mice were fed a high fat diet (HFD) (ssniff EF acc.D12492 (I) mod.) for 16 weeks ad libitum, beginning with 2–3 weeks of age. Experimental animal protocols were approved (number of approval: G-14/067) by the Regierungspraesidium Freiburg, Germany, and all studies conformed to the *Guide for the Care and Use of Laboratory Animals* published by the directive 2010/63/EU of the European Parliament. The composition of the used diets is listed in Additional file 1: Table S1 and S2.

### Metabolic caging

Physiological parameter and metabolic rate of mice was measured in open circuit oxymax chambers, that are a part of the *Comprehensive Lab Animal Monitoring System* (CLAMS, Columbus Instruments, OH). All mice were housed in single cages under a 12 h/12 h light/dark cycle at ~ 23 °C, with food corresponding to the experimental group (either normal chow diet or HFD) and water ad libitum. Prior each setup, mice were weighed. Data from the monitoring cages were automatically collected for 48 h. To obtain reliable results we adapted our experimental conditions to the natural day/night cycle of the mice. As mice were set into the metabolic cages in the morning, we allowed them to acclimate for 24 h in the metabolic cages and used only data from the second day and night.

For indirect calorimetry, O_2_ and CO_2_ concentration of the air entering the chamber was compared to the air leaving the chamber. The sensors for O_2_ and CO_2_ were calibrated using a gas mixture with defined quantities of O_2_, CO_2_ and N_2_. Ambulatory moving of the mice was measured with infrared beams around the cages.

### Glucose and insulin tolerance test

Glucose (GTT) and insulin (ITT) tolerance tests were performed before and at the end of the HFD feeding period. For the GTT, all mice of the experimental groups were fasted over night for 12 h and injected intraperitoneally with a 20% glucose solution (2 g/kg). Blood from tail was collected before and after injection (at 15, 30, 60 and 120 min) and glucose was measured using an ACCU-CHEK Aviva glucometer (Roche Diagnostics). For the ITT, all mice of the experimental groups were fasted for 6 h over day and insulin (I1882, Merck) was injected intraperitoneally (0.75 U/kg). Blood was collected and glucose measured as done for the GTT.

### Measurement of serum and liver cholesterol and triglycerides

Serum samples were taken under non-fasting conditions in the morning at the day of harvest following either high fat or normal chow diet. Serum cholesterol and triglycerides were measured according to the manufacturer’s instructions using the Cholesterol FS (cat. # 113009910021) and Triglyceride FS kit (cat. # 157609910021) from DiaSys, Holzheim, Germany. Briefly, 2 µl of serum sample was mixed with 200 µl of either kit reagent and incubated for 10 min at room temperature. Then, the change in color was measured in an ELISA reader (SpectraMax M2, Molecular Devices, Munich, Germany) at 500 nm against the blank and a standard. Liver cholesterol and triglycerides were determined by incubation of 200 µg total liver protein in 20 µl 0.9% NaCl with 30 µl isopropanol for 10 min at 37 °C with shaking. Afterwards, cell debris was pelleted by centrifugation at 12,000×*g* for 10 min at 4 °C and supernatant was transferred to a new tube following complete evaporation of the liquid at 37 °C, with subsequent measurement using the Cholesterol FS and Triglyceride FS kits as described above.

### Cell culture experiments

Primary hepatocytes were isolated from livers of C57BL/6J or ubiquitous miR-100 overexpressing mice. In brief, 8–10 weeks old male wildtype (n = 7) or miR-100 mice (n = 6) were anesthetized with thiopental (100 µg/g) and a catheter was placed into the *inferior vena cava*. After perfusion with heparin (100 U/100 g), a catheter was placed into the *vena porta hepatica* and all blood was washed out with perfusion medium I (Hanks buffer with 20 mM HEPES, 2 mM EGTA, 0.1% glucose). The dissociation of hepatocytes was achieved by perfusion with collagenase I (100 U/ml) containing medium (Hanks buffer with 20 mM HEPES, 5 mM CaCl_2_) for 10 min. Perfused livers were passed through a 100 µm screening filter and collected by centrifugation at 50×*g* for 2 min. Hepatocytes were re-suspended in Williams Medium E supplemented with 10% FCS, 100 nM dexamethasone, 1 µM insulin and 2 mM glutamine and plated at the desired number in collagen-coated cell culture dishes. After 5–6 h the medium was changed to the medium mentioned above without insulin.

### Oil Red O staining

For Oil Red O staining, cryo preserved liver sections (8 µm) were air dried for 10 min and fixed in 4% formalin for 10 min. After washing for 5 min with tap water, slides were rinsed with 100% propylene glycol for 2 min and stained in Oil Red O solution (0. 5% Oil Red O in 100% propylene glycol) for 25 min at 60 °C. After that, slides were rinsed with tap water for 5 min, washed with ddH2O for 1 min and stained with hematoxylin (#1.05175.0500, modified acc. to Gill II, Merck, Darmstadt, Germany) for 90 s. Then, slides were rinsed in tap water for 2–5 min and mounted with aqueous mounting solution.

### Fatty acid uptake assay

The uptake of fatty acids was measured by incubation of primary hepatocytes with *4,4-difluoro-5,7-dimethyl-4-bora-3α,4α-diaza-s-indacene-3-hexadecanoic acid* (BODIPY FL C16) from Invitrogen. Isolated primary hepatocytes from WT and miR-100 overexpressing mice were cultured on collagen coated glass coverslips in a 12-well tissue culture chamber in Williams Medium E supplemented with 10% FCS, 100 nM dexamethasone and 2 mM glutamine for 24 h. After serum starvation for 3 h, cells were rinsed with 1 × PBS and subsequently incubated for 1 h with 1 × PBS supplemented with DMSO control or BODIPY FL C16 at a final concentration of 200 nmol/L. Afterwards, cells were rinsed three times with ice-cold 1 × PBS and fixed in 4% paraformaldehyde for 10 min. Then, pictures were made from random areas on the coverslip and all well-defined cells were contoured manually using the NIH ImageJ software and the fluorescence intensity was measured. The fluorescence was referenced to blank areas in the field of view and the DMSO control samples.

### Luciferase reporter plasmid activity assay

The luciferase activity assay was done in a modified form as described before (Pankratz et al. 2018) using the Luc-Pair™ Duo-Luciferase Assay Kit 2.0 and the CD36 miTarget™ miRNA 3′ UTR target clone from GeneCopoeia according to the manufactory protocol. Briefly, Huh7 cells were seeded at a density of 1 × 10^4^ cells per well in a white 96 well plate with clear bottom at the day before the transfection. The next day, the cells were transfected with 50 ng of the CD36 3′ UTR reporter plasmid together with the miR-100 precurser molecule (premiR-100) or the miR-100 inhibitory molecule (antimiR-100) or with the respective control molecules (premiR-ctrl or antimiR-ctrl, respectively) using Lipofectamine RNAiMAX. 24 h later, the luciferase reporter activity was measured according to the manufactory protocol and finally, Firefly-luciferase activity was normalized to Renilla-luciferase activity.

### Microarray analysis

A detailed methodology and complete raw data level of the array results were deposited at the Gene Expression Omnibus Database as described under the “Availability of data and materials “section. Briefly, total RNA from liver of C57BL/6J WT and global miR-100 overexpressing mice was isolated using TriPure (Roche Diagnostics) according to manufacturer’s protocol. Gene expression profiles of four independent samples for each condition were assessed using a MoGene-2_0-ST Array (ThermoFisher). We used Partek Genomics Suite software for further analysis (Partek, Inc.). Here, CEL files were imported including control and interrogating probes. Pre-background adjustment was set to adjust for GC Content and probe sequence and RMA background correction was performed. Arrays were normalized using quantile normalization and probe set summarization was done using median polish. Probe values were log2 transformed. In order to identify differentially expressed genes between the groups, we performed ANOVA in Partek.

### MicroRNA and mRNA expression analysis

MiRNA expression was measured as described before (Pankratz et al. 2018) using quantitative taqman-based stem-loop PCR technology (TaqMan MiRNA Assay, Life technologies). The expression was normalized to the expression of the small RNA rnu19. A list of used assays is shown in Additional file 1: Table S3.

For quantitative real-time mRNA expression analysis, 1 µg of total RNA was transcribed using iScript Supermix (iScript, BioRad) according to manufacturer’s instructions as described before (Pankratz et al. 2018) and real-time PCR was performed on a MyIQ cycler (BioRad). The expression of mRNA was normalized to mouse 36B4. For primer sequences please refer to Additional file 1: Table S4.

### Western blot

Liver tissue was lysed in RIPA buffer and protein concentration was determined by Bradford assay (BioRad) following manufacturer’s instructions. Total protein (30 µg per lane) was separated in 9% polyacrylamide-BisTris gels, transferred to PVDF membranes (Bio-Rad), and probed with specific primary antibodies overnight at 4 °C. After that, membranes were incubated with corresponding secondary antibodies for 2 h at room temperature and signals were detected by chemiluminescence using WesternBright reagents (Biozym) in a ChemiDoc MP imaging system (Bio-Rad). The intensity of the bands was analyzed using Image Lab software (Bio-Rad, Version 4.0 build 16). A list of antibodies used is presented in Additional file 1: Table S5.

### Statistical analysis

Data are represented as mean values with SEM. Comparing two groups, significance was calculated by unpaired Student *t*-test with Prism 5 for Windows (GraphPad Software Inc, San Diego, CA, USA). When Gaussian distribution of our data could not be statistically verified, additional non-parametric testing was performed with Mann–Whitney U-tests. One-way ANOVA was used for multiple comparisons of > 2 groups. The Bonferroni post-test for multiple comparisons was used if the P-value for the overall ANOVA comparison was statistically significant. Values of P < 0.05 were considered as statistically significant.

## Results

### Generation of a conditional miR-100 overexpressing transgenic mouse line

**Fig. 1:**
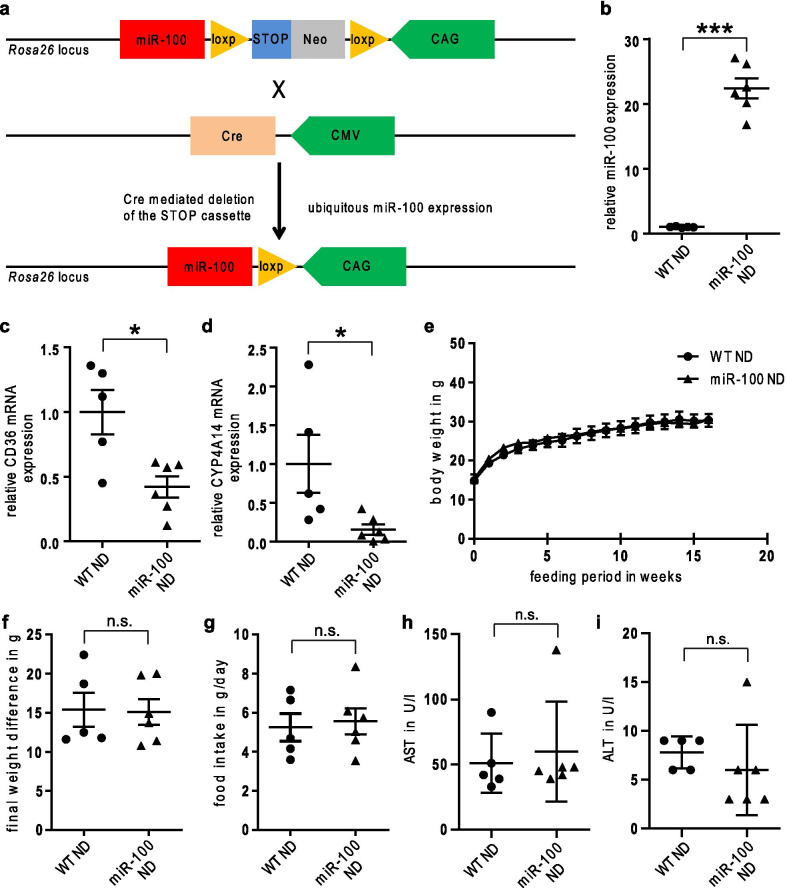
No obvious phenotypic changes in miR-100 overexpressing mice under normal chow diet (ND). **a** The schematic illustration of our miR-100 breeding strategy is shown. **b** The applied breeding scheme resulted in a ~ 22 fold overexpression of miR-100 in liver tissue of transgenic mice as identified using stem-loop based realtime PCR. **c**, **d** CD36 and Cyp4a14 were significantly downregulated in liver tissue of transgenic miR-100 mice compared to WT control as analyzed using quantitative real-time PCR analysis. **e**, **f** The gain in weight was similar in miR-100 mice compared to WT animals under normal chow diet. **g** The food intake in ND was comparable in WT and miR-100 mice. **h**, **i** No significant changes in serum aspartate (AST) and alanine (ALT) transaminases. (n = 5–6 for all experiments) Data represent mean values with SEM. *n.s*. not significant, *P < 0.05 and ***P < 0.001 vs. the corresponding control

To investigate the function of miR-100 in liver metabolism, we developed a conditional miR-100 transgenic mouse line for a gain-of function approach together with a specialized service provider (Genoway, Lyon, France). We chose a conditional approach with expression of a 635 bp sequence coding for miR-100 and stabilizing flanking regions under the control of the CMV early enhancer/chicken beta actin (CCAG) promoter, followed by a loxp-stop-loxp cassette to enable a cell specific expression of miR-100 after breeding with a Cre-deleter strain. The conditional expression using a loxp-stop-loxp setup enabled both the ubiquitous miR-100 overexpression as well as the selective overexpression of miR-100 in specific cell populations. For this study, we decided for a breeding scheme with a CMV-Cre deleter strain (Fig. 1a) to investigate the effects of a ubiquitous miR-100 overexpression on metabolic function. The resulting breeding colony was fertile and showed no phenotypic abnormalities. We could validate the functionality of our construct and found a 22-fold overexpression of miR-100 in liver tissue (Fig. 1b) of transgenic mice compared to wildtype controls.

### Global gene expression analysis in liver tissue of transgenic miR-100 overexpressing mice

Next, we aimed to identify new direct and indirect metabolic target genes regulated by miR-100. Global transcriptome analysis was performed in liver samples of 6-months old transgenic miR-100 mice and wildtype animals as corresponding control. Statistic filtering and testing revealed a list of top nine downregulated genes in miR-100 global overexpressing mice, among them two genes, which are known to be strongly implicated in liver metabolism: *fatty acid translocase /cluster of differentiation 36* (CD36) and *cytochrome P450 4A14* (Cyp4a14) (Table 1) We could verify the downregulation of CD36 and Cyp4a14 in liver tissue of transgenic miR-100 mice on mRNA expression level (Fig. 1c, d).

**Table 1:** Top downregulated genes in liver of miR-100 overexpressing mice

Gene symbol	Name	Fold change	P value
Cyp2b9	Cytochrome P450, family 2, subfamily b, polypeptide 9	− 10.38	0.008
Cyp4a14	Cytochrome P450, family 4, subfamily a, polypeptide 14	− 4.67	0.045
Rps13-ps1	Ribosomal protein S13, pseudogene 1	− 3.34	0.014
Gdpd3	Glycerophosphodiester phosphodiesterase domain containing 3	− 2.59	0.026
Vnn1	Vanin 1	− 2.30	0.044
Cd36	CD36 antigen	− 2.12	0.011
LOC102643284	Adenosylhomocysteinase-like	− 2.10	0.016
Traj43	T cell receptor alpha joining 43	− 2.05	0.009
Cyp17a1	Cytochrome P450, family 17, subfamily a, polypeptide 1	− 2.02	0.028

### Transgenic miR-100 mice show no metabolism-specific phenotypic or genotypic differences compared to wildtype controls under normal chow diet

**Fig. 2:**
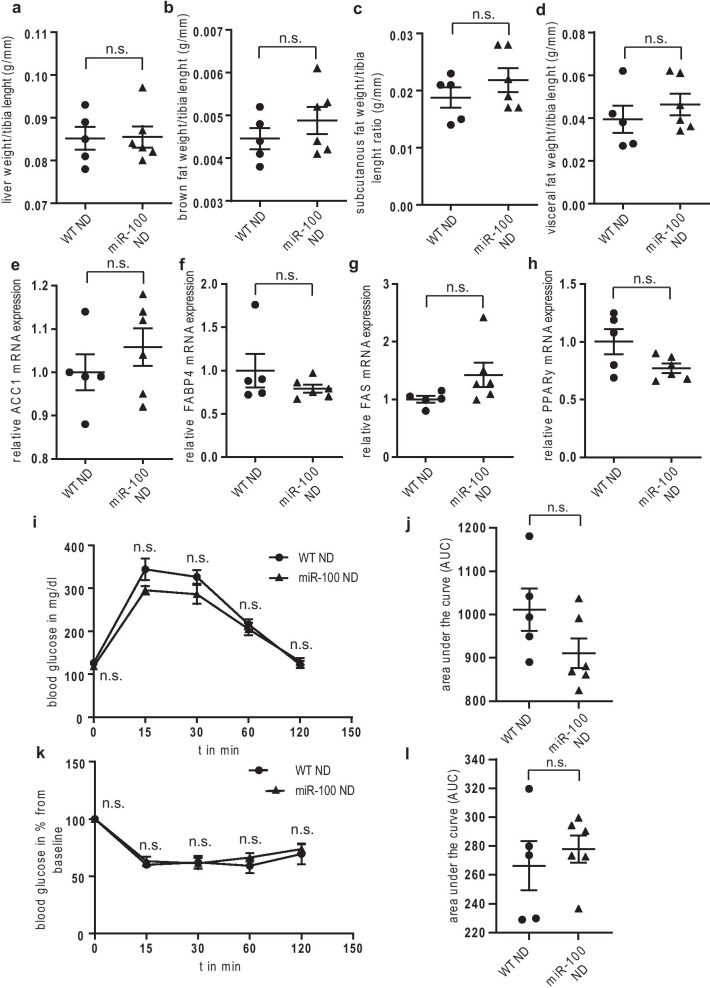
No metabolic changes under normal chow diet (ND) in miR-100 mice. **a**–**d** There was no difference in the weight of liver (**a**), brown fat tissue (**b**), subcutaneous fat (**c**) and visceral fat (**d**) at the end of the 16 weeks ND feeding period (age 18–19 weeks) in miR-100 overexpressing mice in comparison to the wildtype controls. **e**–**h** The expression of lipogenic genes in the liver of miR-100 mice was not significantly altered as seen by quantitative real-time PCR analysis compared to WT animals. **i**, **j** The intraperitoneal glucose tolerance test at the end of the feeding period revealed no difference in WT and miR-100 mice. **k**, **l** The intraperitoneal insulin tolerance test at the end of the feeding period showed no change in miR-100 mice compared to wildtype controls. (n = 5–6 for all experiments) Data represent mean values with SEM. *n.s.* not significant vs. the corresponding control

Based on the results of the global transcriptome analysis, we investigated potential metabolic-specific phenotypic and genotypic changes in the transgenic, global miR-100 overexpressing mouse line under normal chow diet (ND) in a physiological setting. Body weight did not differ between the groups over a feeding period of 16 weeks under ND (Fig. 1e, f). Accordingly, we could not find any significant difference in food intake (determined in metabolic cages over 48 h, Fig. 1g). Additionally, the level of serum aspartate transaminase (AST) and alanine transaminase (ALT) of transgenic miR-100 mice were comparable to wildtype controls, suggesting no effect of miR-100 overexpression on normal liver function under physiologic condition (Fig. 1h, i, respectively). Next, we analyzed the whole body composition and could not find any differences in liver mass and fat deposition (Fig. 2a–d), overall indicating no significant impact of miR-100 on metabolic processes without an additional stressor. This conclusion was confirmed when we measured the expression of the key genes of fatty acid metabolism such as ACC1, FABP4, FAS and PPARγ (Fig. 2e–h). None of these parameters showed a significant regulation after miR-100 overexpression. Finally, we performed glucose and insulin tolerance tests in mice 18–19 weeks of age fed with normal chow diet and again, found no significant changes if miR-100 was globally overexpressed (Fig. 2i–l).

### MiR-100 overexpression attenuates HFD-induced obesity and liver steatosis in mice

**Fig. 3:**
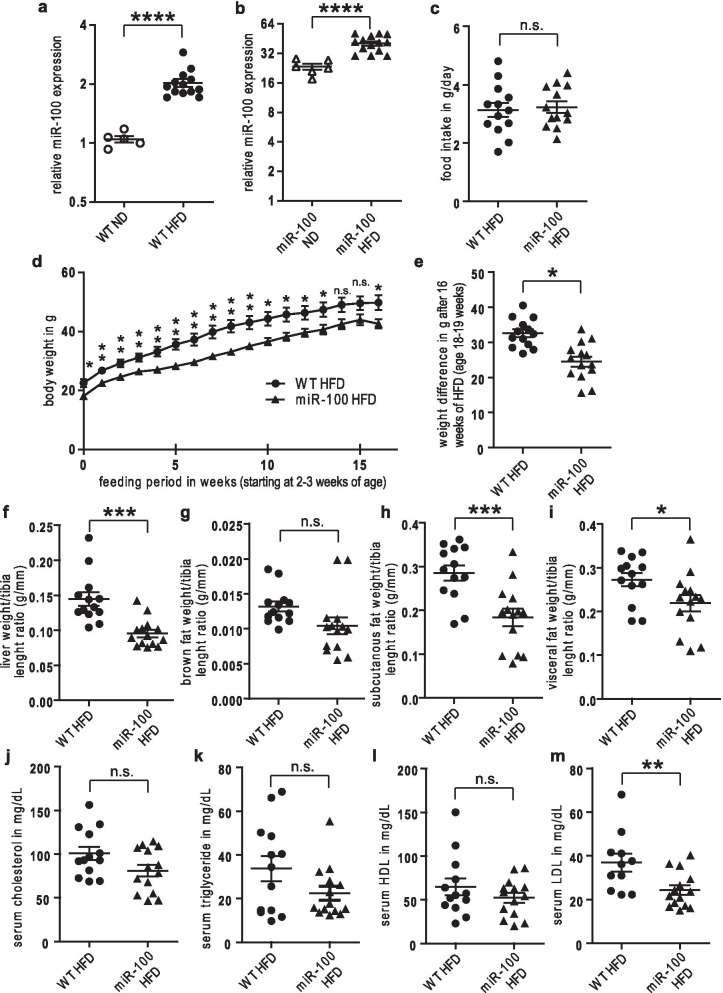
Reduced weight gain in miR-100 mice under 16 weeks high fat diet feeding. **a**, **b** High fat diet (HFD) feeding (**b**) (n = 6–14) increased the expression of miR-100 in wildtype and miR-100 overexpressing mice compared to normal chow diet (ND) (**a**) (n = 5–13). **c** The food intake in the HFD group was similar between WT and miR-100 mice. **d** The gain in weight during the 16 weeks of high fat diet feeding was reduced in miR-100 animals. **e** The final weight (age 18–19 weeks) was significantly different in miR-100 mice compared to WT. **e**–**h** The weight of liver (**f**), subcutaneous fat (**h**) and visceral fat (**i**) was significantly lower at the end of the HFD feeding period but not the amount of brown fat tissue (**g**). **j**–**m** Total serum cholesterol (**j**), triglyceride (**k**) and HDL (**l**) levels were not reduced, whereas LDL (**m**) showed a significant difference in miR-100 overexpressing mice. (n = 13–14) Data represent mean values with SEM. *n.s.* not significant, *P < 0.05, **P < 0.01 and ***P < 0.001 vs. the corresponding control

We next investigated the impact of this miRNA under pathophysiological condition by adding a high fat diet (HFD) as a metabolic stressor. A cohort of 2–3 weeks old male wildtype and transgenic miR-100 mice was fed ad libitum over a period of 16 weeks using a diet containing 60% of energy gained from fat. Wildtype mice under HFD showed a twofold enhanced miR-100 expression compared to ND wildtype controls (Fig. 3a) and also transgenic miR-100 mice under HFD exhibited a twofold upregulation of miR-100 when compared to transgenic mice fed with ND (Fig. 3b).

Although the food intake was comparable between transgenic mice and wildtype controls as determined by housing the mice in metabolic cages (48 h at beginning and at the end of the study, Fig. 3c), we observed a significantly reduced overall weight gain in transgenic miR-100 mice (Fig. 3d, e). Analysis of the body composition revealed a significantly reduced liver mass as well as subcutaneous and visceral adipose tissue (Fig. 3f, h, i). The content of brown fat was unaffected (Fig. 3g).

**Fig. 4:**
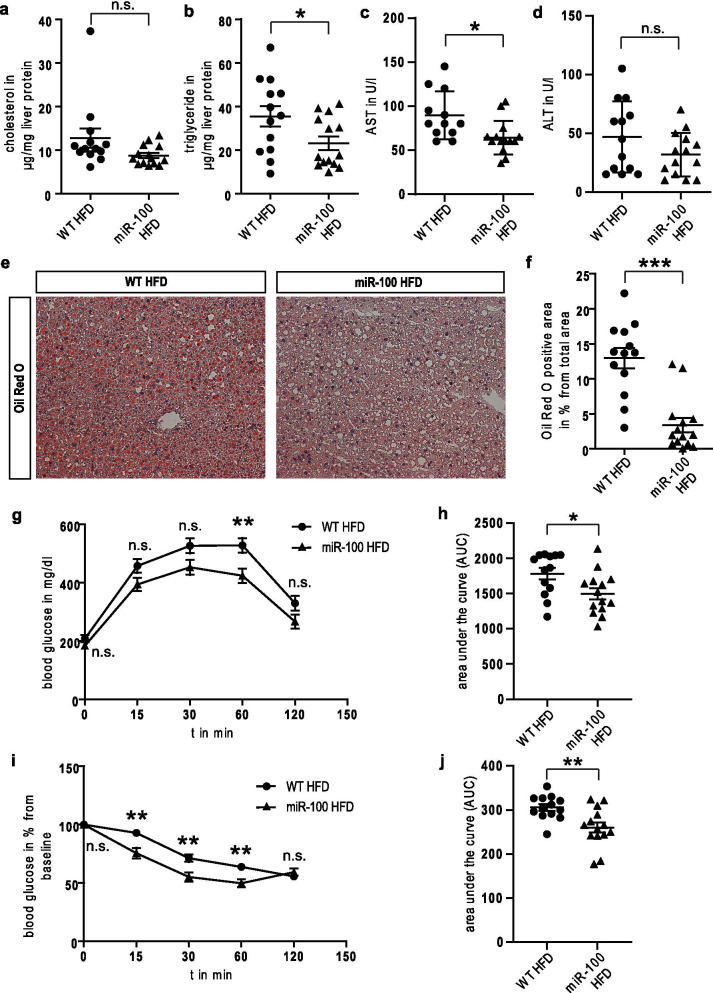
Reduced hepatic fat accumulation in miR-100 mice under 16 weeks high fat diet feeding. **a**, **b** The level of liver cholesterol (**a**) was not significantly reduced, whereas triglycerides (**b**) were diminished in miR-100 animals. **c**, **d** Serum aspartate transaminase (AST) activity (**c**) was reduced in miR-100 overexpressing mice, whereas no difference was detectable in serum alanine transaminase (ALT) (**d**). **e** Representative pictures of cryo preserved liver sections (8 µm) stained for Oil Red O are shown. **f** The quantification of the Oil Red O positive areas was done using Image J software. Transgenic miR-100 mice displayed an improved glucose tolerance in glucose tolerance test (GTT) (**g**, **h**) as well as insulin sensitivity in insulin tolerance test (ITT) (**I**, **j**) compared to wildtype animals. (n = 13–14 for all experiments) Data represent mean values with SEM. *n.s.* not significant, *P < 0.05, **P < 0.01 and ***P < 0.001 vs. the corresponding control

Based on the observed difference in fat disposition, we next examined whether a global miR-100 overexpression affected cholesterol- and triglyceride homeostasis. We did not find any significant changes in serum cholesterol, triglyceride and HDL (Fig. 3j–l), but levels of LDL cholesterol were decreased (Fig. 3m). However, whereas the cholesterol content in the transgenic liver samples was not affected, we observed a reduced content of triglycerides extracted from liver tissue of miR-100 overexpressing mice (Fig. 4a, b). Moreover, we could detect a significantly reduced serum AST in miR-100 mice, whereas ALT did not differ compared to WT animals (Fig. 4c, d). Additionally, we found less Oil Red O positive staining (Fig. 4e, f) and reduced fat accumulation as quantified by HE staining (Additional file 2: Fig. S1) in miR-100 mice, indicating an influence of this miRNA in the regulation of fatty acid storage in hepatocytes.

In good correspondence to the observed effects on body weight and liver steatosis, we found that transgenic miR-100 overexpressing mice displayed an improved GTT (Fig. 4g, h) as well as ITT (Fig. 4i, j) compared to wildtype animals under a HFD at the end of the feeding period, whereas no significant difference in GTT (Additional file 2: Fig. S2a, b, e, f) and ITT (Additional file 2: Fig. S2c, d, g, h) could be detected at the start of ND (Additional file 2: Fig. S2a–d) and HFD (Additional file 2: Fig. S2e–h).

### HFD mediated induction of fat metabolism is attenuated in miR-100 mice

**Fig. 5:**
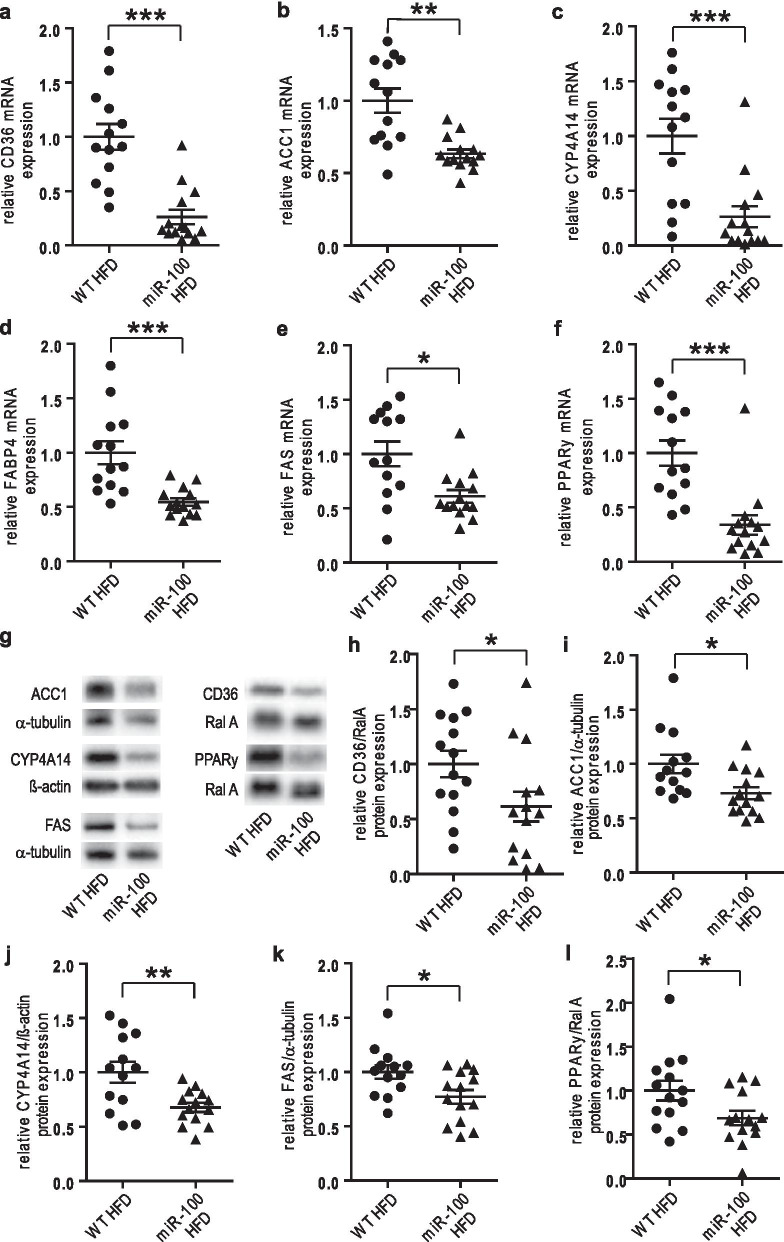
Hepatic lipogenic genes and proteins are decreased in miR-100 mice after 16 weeks of HFD. **a**–**f** The lipogenic gene program was robustly suppressed in miR-100 transgenic animals under HFD compared to WT as revealed by quantitative real-time PCR analysis. **g** Representative Western blots of key enzymes of lipogenesis (ACC1, FAS and PPARγ) and the potential miR-100 targets (CD36 and CYP4A14) are demonstrated. **h**–**l** The quantification of Western blots seen in (**g**) is shown. (n = 13–14 for all experiments) Data represent mean values with SEM. *P < 0.05, **P < 0.01 and ***P < 0.001 vs. the corresponding control

We went on to analyze the lipogenic gene profile in transgenic miR-100 and wildtype mice in the pathophysiological setting of a HFD. In contrast to our findings under ND, miR-100 overexpression under metabolic stress significantly reduced the expression of the key genes and proteins of fatty acid storage and -metabolism such as CD36, ACC1, FABP4, FAS and PPARγ in liver tissue (Fig. 5a, b, d–i, k, l). Additionally, we also found a significant attenuation of Cyp4A14 expression in miR-100 overexpressing mice under HFD, which is in good correspondence to our finding in the initial screening experiment (Fig. 5c, j).

**Fig. 6:**
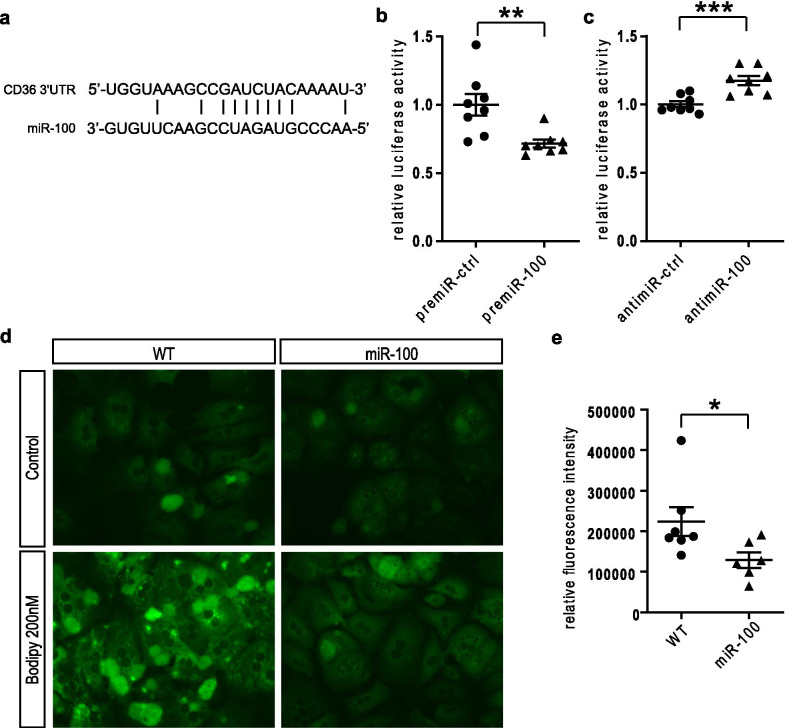
MiR-100 overexpression attenuates lipid accumulation in primary cultured hepatocytes. **a** The illustration shows the potential binding site of miR-100 in the 3′UTR of CD36. **b** The luciferase activity was reduced when the CD36 3′UTR plasmid and miR-100 precurser molecules (premiR-100) were overexpressed (n = 8). **c** The luciferase activity was induced when the CD36 3’UTR plasmid was overexpressed together with the miR-100 inhibitory molecule antimiR-100 (n = 8). **d** Exemplary fluorescence images of BODIPY FL C16 uptake in primary cultured mouse hepatocytes isolated from wildtype and miR-100 overexpressing mice are shown (Magnification: ×400). The hepatocytes were treated with DMSO control or BODIPY FL C16 at 200 nM for 1 h. **e** The quantification of BODIPY FL C16 revealed a reduced uptake into hepatocytes isolated from miR-100 overexpressing mice. (n = 6–7) Data represent mean values with SEM. *P < 0.05, **P < 0.01 and ***P < 0.001 vs. the corresponding control

Interestingly, although CD36 is not listed as a potential direct miR-100 target in common target site prediction tools such as Targetscan (Friedman et al. 2009; Grimson et al. 2007; Lewis et al. 2005) and miRBase (Kozomara et al. 2019), we found potential binding sites for miR-100 in the 3′UTR of the mouse CD36 mRNA sequence (Fig. 6a). To confirm a direct regulation of CD36 by miR-100, a dual-luciferase reporter assay using the Luc-Pair™ Duo-Luciferase assay system was performed. Huh7 cells were co-transfected with both the plasmid containing the sequence of the CD36 3’UTR and premiR-100 or antimiR-100 or their respective control plasmids. Indeed, our results showed a modulation of CD36 through miR-100 by interacting with the 3′UTR of CD36, for the first time validating CD36 as a direct miR-100 target (Fig. 6b, c).

### Fatty acid uptake is reduced in cultured miR-100 overexpressing primary hepatocytes

Our findings of a direct regulation of CD36 by miR-100 in combination with lower triglyceride content in liver tissue of miR-100 transgenic mice subsequently resulted in the investigation of the fatty acid uptake capacity of hepatocytes either isolated from the transgenic global miR-100 mice or wildtype animals. Primary hepatocytes were treated with the fluorescently labelled oleic acid analogue BODIPY FL C16 and incorporation of this fatty acid in primary hepatocytes was monitored using fluorescence microscopy. We found a reduced uptake of BODIPY reagent in miR-100 overexpressing hepatocytes (Fig. 6d, e), supporting the hypothesis that miR-100 impairs the uptake and in consequence the storage of fatty acids in the liver by regulating CD36.

### MiR-100 mice demonstrate increased energy expenditure

**Fig. 7:**
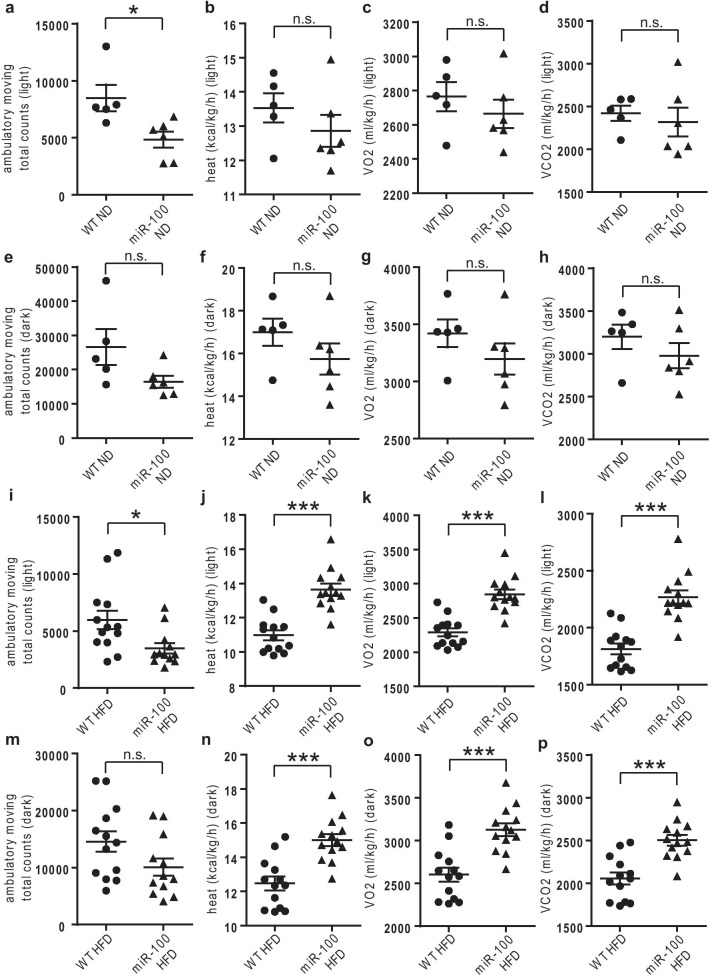
MiR-100 mice demonstrate increased energy expenditure under high fat diet but not normal chow diet. **a**–**h** At the end of the ND feeding period, the *comprehensive laboratory animal monitoring system* (CLAMS) measured ambulatory moving (**a**, **e**), heat generation (**b**, **f**), O_2_ consumption (**c**, **g**) and CO_2_ production (**d**, **h**). The graphs indicate the average during the day (**a**–**d**) and night (**e**–**f**). **i**–**p** At the end of the HFD feeding period, the CLAMS measured ambulatory moving (**I**, **m**), heat generation (**j**, **n**), O_2_ consumption (**k**, **o**) and CO_2_ production (**l**, **p**). The graphs indicate the average during the day (**i**–**l**) and night (**m**–**p**). (ND n = 5–6 and HFD n = 13) Data represent mean values with SEM. *n.s.* not significant, *P < 0.05 and ***P < 0.001 vs. the corresponding control

Additionally to the expression analysis, we investigated the physical activity and energy expenditure (heat) of wildtype and miR-100 mice under ND and HFD conditions, using the *comprehensive laboratory animal monitoring system* (CLAMS). Surprisingly, we found that miR-100 overexpressing mice showed significantly less total ambulatory moving under ND and HFD during the day (Fig. 7a, i), which is in strong contrast to our finding that miR-100 mice develop less weight gain (Fig. 3d, e) but eat similar (Fig. 3c). The ambulatory moving in the dark (Fig. 7e, m) showed no significant difference. Possibly, the observed discrepancy between the decreased weight gain and less moving combined with comparable food intake in HFD fed miR-100 animals may be caused by an increased internal heat production. And indeed, indirect calorimetry analysis using the CLAMS system revealed that the heat production was significantly increased in HFD fed transgenic miR-100 mice compared to wildtype controls during both, day and night (Fig. 7j, n). Correspondingly, the total volume of oxygen consumed (VO2, Fig. 7k, o) and the carbon dioxide produced (VCO2, Fig. 7l, p) were significantly increased in our transgenic miR-100 mice under HFD feeding during day and night. Under ND conditions, there were no significant changes in heat production, O_2_ consumption or CO_2_ production between wildtype and miR-100 mice detectable (Fig. 7b–d during the day and Fig. 7f–h during the night).

### Reduced inflammation in the liver of HFD fed miR-100 mice

Obesity is strongly associated with chronic inflammatory processes and when examining inflammation-related genes such as IL-1β as well as TNF-α we found a significantly attenuated expression in liver tissue of transgenic miR-100 mice that underwent a HFD for 16 weeks (Additional file 2: Fig. S3a, b). Additionally, the expression of the oxidative stress marker Vanin-1 was also reduced in the liver of miR-100 overexpressing mice under HFD conditions (Additional file 2: Fig. S3c).

## Discussion

Disproportional nutrient intake and metabolic dysregulation can result in obesity and hepatic steatosis, which occurs in 10–25% of people worldwide and are major risk factors for the development metabolic and cardiovascular diseases (Wilson et al. 2016). In the current study, we describe a transgenic mouse strain with a ubiquitous overexpression of miR-100, which is partially protected against these harmful sequelae of a HFD. MiR-100 overexpressing mice gain less excessive weight and develop less visceral and subcutaneous fat tissue than wildtype animals. In addition, they exhibit a lower liver mass due to less fat accumulation, accompanied by less hepatic inflammation, an improved glucose tolerance and increased insulin sensitivity. The metabolic phenotype under a normal diet remains unaffected by miR-100.

Our findings support previous reports linking miR-100 to metabolic diseases: Pek et al. found miR-100 to be downregulated in blood and visceral fat of patients with obesity and type 2 diabetes (Pek et al. 2016) and showed that miR-100 inhibition results in an increased adipocyte differentiation whereas an inhibition of miR-100 reduces the differentiation potential of 3T3-L1 cells. In addition, Ortega et al. showed that miR-100 expression decreased during adipocyte differentiation. The known regulation of mTOR-signaling by miR-100 was presumed to be the responsible mechanism (Ortega et al. 2010).

In contrast to our findings of protective effects of miR-100 overexpression on liver steatosis, most other studies address the inhibition of liver specific miRNAs. Antisense treatment against miR-122 as the most abundant miRNA in the liver, has been shown to prevent hepatic steatosis (Esau et al. 2006) and loss of miR-22 reduced fat mass gain and prevented HFD induced dyslipidemia by diminishing the expression of genes involved in lipogenesis (Diniz et al. 2017). A protective role of miR-155 in non-alcoholic hepatosteatosis was described by Miller et al., who found an increased hepatic steatosis in miR-155 knockout animals under HFD, associated with increased liver weight and serum VLDL/LDL level (Miller et al. 2013). Our study now adds miR-100 as another potential protective “metabomiR” to this study field. Besides the possible regulatory function of miRNAs in NAFLD, another aspect could be the use as biomarker to diagnose the different stages of NAFLD as liver biopsy is to date the gold standard diagnosis method. Specifically, miR-122 and miR-34a serum level have been shown to correlate with among others hepatic inflammation and fibrosis stage (Cermelli et al. 2011; Jampoka et al. 2018; Liu et al. 2018a)*.* However, miR-100 is not exclusively expressed in liver tissue and whether this microRNA is differentially expressed in serum of patients suffering from NAFLD needs to be further elucidated.

In our current study, global gene expression analysis in hepatic tissue from miR-100 overexpressing and wildtype mice fed with ND reveals two other genes involved in lipid metabolism and storage among the top 9 downregulated genes: the *scavenger receptor and fatty acid transporter CD36* (CD36) and the *cytochrome P450 omega-hydroxylase 4A14* (Cyp4a14). CD36 belongs to the class B of the scavenger receptor family and is able to bind a variety of molecules, including oxidized LDL, phospholipids, collagen and long chain fatty acids (Endemann et al. 1993; Febbraio et al. 2002; Liu et al. 2018b). It is highly expressed in cardiomyocytes, skeletal muscle cells, adipocytes and macrophages (Ibrahimi et al. 1999) and at markedly lower levels in hepatocytes, but hepatic expression increases rapidly under lipid rich diets, hepatic steatosis and in nonalcoholic fatty liver disease (NAFLD) (Greco et al. 2008; Miquilena-Colina et al. 2011). Increased CD36 expression has been shown to correlate with dyslipidemia (Koonen et al. 2007) and inflammatory hepatic stress (Liu et al. 2018b) as well as to enhance the susceptibility to develop hepatic steatosis and NAFLD (Sheedfar et al. 2014; Steneberg et al. 2015). In rodents, the hepatocyte specific disruption of CD36 attenuates the development of fatty liver diseases and insulin resistance induced by a HFD (Bonen et al. 2015; Wilson et al. 2016). In mouse models of metabolic disease, hepatic overexpression of CD36 increases liver triglyceride storage (Koonen et al. 2007), whereas the liver specific disruption of CD36 attenuates the development of fatty liver under HFD (Wilson et al. 2016). These findings are in good correspondence with our own observations, where the direct regulation of CD36 by miR-100 leads to a decrease in long chain fatty acid uptake in primary miR-100 overexpressing hepatocytes.

Cyp4A14 belongs to the Cyp4A subfamily which forms together with 17 other members the Cyp4 family of cytochrome P450, which catalyzes the omega-hydroxylation of saturated, branched chain and unsaturated fatty acids. In mice, Cyp4A14 expression has been shown to be induced by PPARα (Zhang and Klaassen 2013) and to be increased in HFD-induced nonalcoholic fatty liver disease (Patsouris et al. 2006). Inhibition of Cyp4A14 results in the attenuation of insulin resistance and apoptosis in diabetic mice (Park et al. 2014) and Zhang et al. found a reduced hepatic lipid accumulation accompanied with a diminished expression of liver Fat/CD36 expression (Zhang et al. 2017) which is in good correspondence to our findings in the transgenic miR-100 mouse strain.

Despite pronounced effects of miR-100 on its direct target mTOR in cardiovascular cells as shown by our group (Pankratz et al. 2018), these metabolic key enzyme seems to play a minor role as a miR-100 target in hepatic cells as our screening experiment does not reveal mTOR as a regulated gene by miR-100 in liver tissue.

Interestingly, while the changes in metabolic gene expression are already detectable under normal diet, the changes in the metabolic phenotype of miR-100 overexpressing mice are only observed under HFD, arguing for the dependency of miR-100 mediated effects on this metabolic stressor. In addition to the observed effects on the direct miR-100 targets described above, miR-100 overexpressing mice show a downregulation of several additional key mediators of fatty acid storage and metabolism. Especially the expression of the master regulator of adipogenesis PPARγ (Lefterova et al. 2014) is markedly decreased, along with the reduction of its downstream target genes ACC1, FABP4 and FAS, potentially contributing to the observed effects on lipogenesis and fatty acid uptake. Although the precise regulation of these metabolic mediators is not investigated in our study, PPARγ has been shown to be repressed by downregulation of CD36 (Demers et al. 2008; Rodrigue-Way et al. 2014), which is in good correspondence with our findings.

As an interesting detail, metabolic cage phenotyping of our transgenic mice shows that the difference in weight gain is independent of nutrient intake and movement. Indeed, miR-100 overexpressing mice gain less weight despite a more sedentary behavior with less ambulatory movement, but a higher oxygen consumption, which can be caused by an uncoupling from oxidative phosphorylation in other tissues in transgenic miR-100 mice or by a minor contribution of the locomotor activity to the total energy expenditure compared to the basal metabolism. However, further investigation has to be performed to clarify the coherences.

The observed effects of diminished serum triglycerides of our transgenic miR-100 mice following HFD in the current study fits very well to our other findings on miR-100 dependent effects on different aspects of metabolic syndrome, but it is in partial contrast to our previous study linking miR-100 to chronic vascular inflammation (Pankratz et al. 2018). Here, we observed an overall reduction of serum triglycerides and cholesterol following pharmacological overexpression of miR-100 under high cholesterol diet. However, both studies differ in regard to diet (high fat vs. high cholesterol diet), feeding time (8 vs. 16 weeks), overexpression approach (transgenic vs. pharmacological) and genetic background (C57BL/6J and LDLR-/-).

It has to be noticed that serum cholesterol and triglycerides were determined under non-fasting conditions, nevertheless, it has been shown that many metabolites are not drastically affected by fasting status (Langsted and Nordestgaard 2019; Stevens et al. 2019).

## Conclusions

In summary, we add miR-100 as a novel “metabomiR” with a protective function in metabolic syndrome by reducing weight gain, liver steatosis and hypertriglyceridemia. In detail, miR-100 reduces the expression of lipogenic enzymes and directly interacts with CD36, preventing excessive lipid accumulation in the liver. Interestingly, transgenic miR-100 mice display less weight gain despite concurrent reduced ambulatory movement. Further studies addressing the underlying effects of this observation are warranted and could offer new insights into the pathogenesis of metabolic syndrome and possible miRNA-based therapeutic strategies.

## Supplementary Information

**Additional file 1: Table S1.** Normal chow diet (ND) composition. The ND (#3437) was purchased fro, LIBA NAFAG, Kaiseraugst, Switzerland. **Table S2.** High fat diet (HFD) composition. The HFD (ssniff EF acc. D12492(I) mod.) was purchased from ssniff Spezialdiäten GmbH, Soest, Germany. **Table S3.** List of Taqman assays used for quantitative stem-loop PCR analysis and miRNA molecules used for transfection. **Table S4.** List of primer sets used for SYBR green based quantitative real-time PCR analysis. **Table S5.** List of primary and secondary antibodies used for Western blot analysis.

**Additional file 2: Figure S1.** Fat accumulation under high fat diet is reduced in miR-100 mice compared to wildtype. (a) Representative pictures of paraffin preserved liver sections (8 µm) stained for HE are shown. (b) The quantification of fat accumulation (white areas) was done using Image J software. (n = 13–14) Data represent mean values with SEM. ***P < 0.001 vs. the corresponding control. **Figure S2.** No changed glucose tolerance and insulin sensitivity in miR-100 mice at start of the 16 weeks normal chow and high fat diet feeding period. (a–d) MiR-100 mice showed no altered glucose tolerance in the GTT (a, b) and insulin sensitivity in the ITT (c, d) compared to wildtype animals fed normal chow diet before the feeding period started (age 2–3 weeks, n = 5–6). (e–h) MiR-100 mice showed no altered glucose tolerance in the GTT (e, f) and insulin sensitivity in the ITT (g, h) compared to wildtype animals before the high fat feeding period started. (age 2–3 weeks, n = 13–14) Data represent mean values with SEM. n.s. = not significant, *P < 0.05, **P < 0.01 and ***P < 0.001 vs. the corresponding control. **Figure S3.** Reduced inflammation in the liver of miR-100 mice compared to wildtype after HFD feeding. (a, b) Quantitative real-time PCR analysis at the end of the HFD feeding period revealed a significant reduction of the inflammatory marker genes IL-1β and TNFα in miR-100 livers. (c) The expression of the oxidative stress marker Vnn1 is also significantly reduced in the liver of miR-100 overexpressing mice compared to wildtype. (age 18–19 weeks, n = 13–14) Data represent mean values with SEM. **P < 0.01 and ***P < 0.001 vs. the corresponding control.

## Data Availability

The datasets supporting the conclusion of this article are included within the article and Additional files 1 and 2. Microarray data are available in Gene Expression Omnibus Database at https://www.ncbi.nlm.nih.gov/geo/, and can be accessed with Accession Number GSE167603.

## Abbreviations

36B4: Acidic ribosomal phosphoprotein P0
3′UTR: Three prime untranslated region
ACC1: Acetyl-CoA carboxylase
antimiR: MicroRNA inhibitor molecule
BMI: Body mass index
CD36: Fatty acid translocase/cluster of differentiation 36
CLAMS: Comprehensive lab animal monitoring system
CMV: Cytomegalovirus
CO_2_: Carbon dioxide
ctrl: Control
Cyp4A14: Cytochrome P450 omega-hydroxylase 4A14
DMSO: Dimethyl sulfoxide
FABP4: Fatty acid-binding protein 4
FAS: Fatty acid synthase
FCS: Fetal calf serum
GOT: Glutamate–oxaloacetate transaminase
GPT: Glutamate-pyruvate transaminase
GTT: Glucose tolerance test
HDL: High-density lipoprotein
HFD: High fat diet
ITT: Insulin tolerance test
LDL: Low-density lipoprotein
miR: MicroRNA
mRNA: Messenger ribonucleic acid
mTOR: Mechanistic target of rapamycin
NaCl: Sodium chloride
NAFLD: Nonalcoholic fatty liver disease
N_2_: Dinitrogen
ND: Normal chow diet
O_2_: Dioxygen
PBS: Phosphate buffered saline
PCR: Polymerase chain reaction
PPARα: Peroxisome proliferator-activated receptor alpha
PPARγ: Peroxisome proliferator-activated receptor gamma
premiR: Precursor microRNA
PVDF: Polyvinylidene difluoride
Ral A: Ras-related protein Ral-A
RNA: Ribonucleic acid
VLDL: Very-low-density lipoprotein
WT: Wildtype
