# Supplementary material for: MiR-100 overexpression attenuates high fat diet induced weight gain, liver steatosis, hypertriglyceridemia and development of metabolic syndrome in mice
Source: Mol Med. 2021 Sep 6;27:101. doi: 10.1186/s10020-021-00364-6 (PMC8422764; doi:10.1186/s10020-021-00364-6)
Supplement: Supplementary file 1 — Additional file 1: Table S1. Normal chow diet (ND) composition. The ND (#3437) was purchased fro, LIBA NAFAG, Kaiseraugst, Switzerland. Table S2. High fat diet (HFD) composition. The HFD (ssniff EF acc. D12492(I) mod.) was purchased from ssniff Spezialdiäten GmbH, Soest, Germany. Table S3. List of Taqman assays used for quantitative stem-loop PCR analysis and miRNA molecules used for transfection. Table S4. List of primer sets used for SYBR green based quantitative real-time PCR analysis. Table S5. List of primary and secondary antibodies used for Western blot analysis. [file 10020_2021_364_MOESM1_ESM.docx]

**
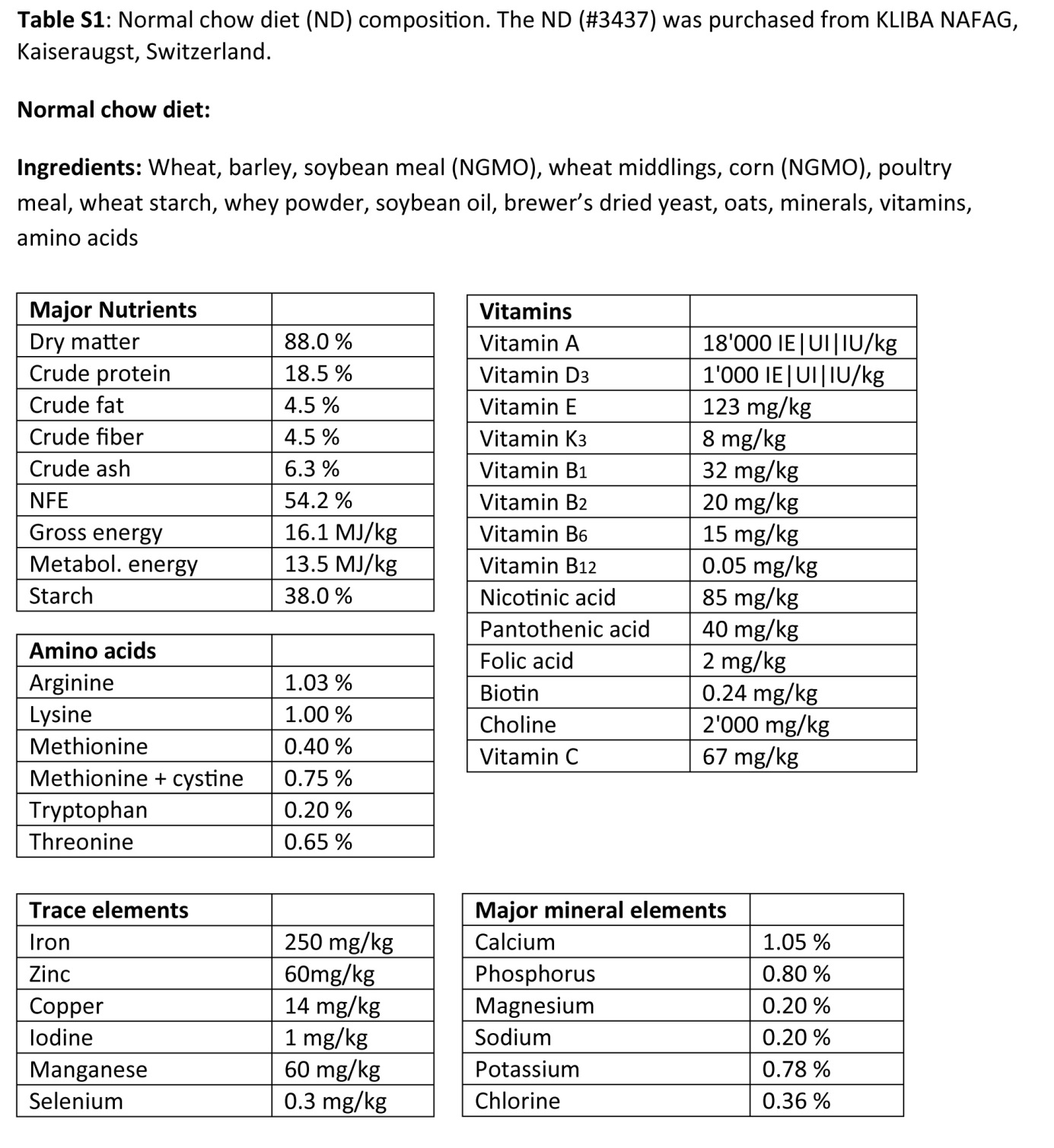
**

**
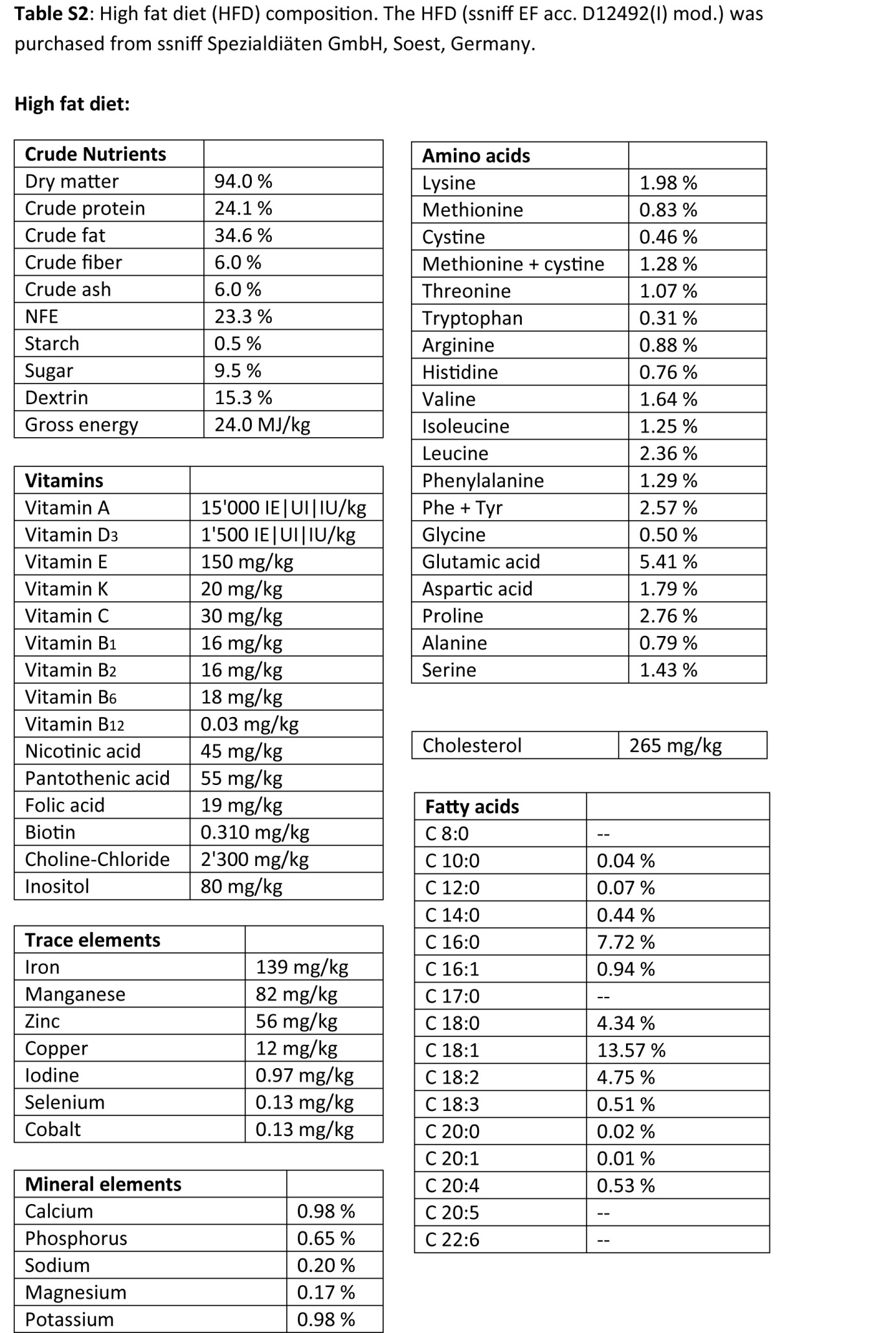
**

**Table S3**: List of Taqman assays used for quantitative stem-loop PCR analysis and miRNA molecules used for transfection.

| **Taqman Assay** |  |  |  |
| --- | --- | --- | --- |
| **Name** | **Assay ID** | **Kit Part No.** | **Company** |
| mmu/hsa-miR-100 | 000437 | 4440887 | Life technologies |
| Rnu19 | 001003 | 4440887 | Life technologies |

| **miRNA** |  |  |  |
| --- | --- | --- | --- |
| **Name** | **Cat #** | **ID #** | **Company** |
| premiR-cont. | AM17110 | N/A | Life technologies |
| premiR-100 | AM17100 | PM10188 | Life technologies |
| antimiR-cont. | AM17010 | N/A | Life technologies |
| antimiR-100 | AM17000 | AM10188 | Life technologies |

**Table S4**: List of primer sets used for SYBR green based quantitative real-time PCR analysis.

| **Primer** |  |  |  |
| --- | --- | --- | --- |
| **Gene** | **Species** | **Forward** | **Reverse** |
| ACC1 | mouse | 5’-ACCGTCTGCTGGGAAGTTAAT-3’ | 5’-GATGCAGCCAGATTCTACAGC-3’ |
| CD36 | mouse | 5’-CCATTCCTCAGTTTGGTTCC-3’ | 5’-TGCATTTGCCAATGTCTAGC-3’ |
| CYP4A14 | mouse | 5’-TGAATTGCTGCCAGATCCCAC-3’ | 5’-GTTCAGTGGCTGGTCAGAGTT-3’ |
| FABP4 | mouse | 5’-GATGAAATCACCGCAGACGACA-3’ | 5’-ATTGTGGTCGACTTTCCATCCC-3’ |
| FAS | mouse | 5’-CCCTTGATGAAGAGGGATCA-3’ | 5’-ACTCCACAGGTGGGAACAAG-3’ |
| IL-1β | mouse | 5’-CAACCAACAAGTGATATTCTCCAT-3’ | 5’-GATCCACACTCTCCAGCTGCA-3’ |
| PPARγ | mouse | 5’-GCCCTTTGGTGACTTTATGGA-3’ | 5’-GCAGCAGGTTGTCTTGGATG-3’ |
| TNFα | mouse | 5’-ACCCCTTTATTGTCTACTCCTC-3’ | 5’-GTCCCAGCATCTTGTGTTTC-3’ |
| Vnn-1 | mouse | 5’-TTATGCCTTTGGAGCCTTTG-3’ | 5’-AGGGAAGACATACCGGGTTC-3’ |
| 36B4 | mouse | 5’-AAGCGCGTCCTGGCATTGTCT-3’ | 5’-CCGCAGGGGCAGCAGTGGT-3’ |

**Table S5**: List of primary and secondary antibodies used for Western blot analysis.

| **Primary antibodies** |  | | |  | |  | |
| --- | --- | --- | --- | --- | --- | --- | --- |
| **Target** | **Host** | | | **Clone ID** | | **Company** | |
| α-tubulin | mouse | | | DM1A | | Sigma-Aldrich | |
| ACC1 | rabbit | | | polyclonal | | Cell Signaling | |
| β-actin | rabbit | | | polyclonal | | abcam | |
| CD36 | rabbit | | | EPR6573 | | abcam | |
| CYP4A | mouse | | | E-6 | | Santa Cruz | |
| FAS | rabbit | | | C20G5 | | Cell Signaling | |
| PPARγ | mouse | | | B-5 | | Santa Cruz | |
| RalA | mouse | | | 8/Ral A | | BD Transduction Laboratories | |
| **Secondary antibodies** | |  |  | |  | |  |
| **Target** | | **Host** | **Clone ID** | | **Company** | | **Label** |
| Rabbit IgG | | goat | - | | Thermo Scientific | | HRP |
| Mouse IgG | | goat | - | | R&D Systems | | HRP |
