# Supplementary material for: MiR-100 overexpression attenuates high fat diet induced weight gain, liver steatosis, hypertriglyceridemia and development of metabolic syndrome in mice
Source: Mol Med. 2021 Sep 6;27:101. doi: 10.1186/s10020-021-00364-6 (PMC8422764; doi:10.1186/s10020-021-00364-6)
Supplement: Supplementary file 2 — Additional file 2: Figure S1. Fat accumulation under high fat diet is reduced in miR-100 mice compared to wildtype. (a) Representative pictures of paraffin preserved liver sections (8 µm) stained for HE are shown. (b) The quantification of fat accumulation (white areas) was done using Image J software. (n = 13–14) Data represent mean values with SEM. ***P < 0.001 vs. the corresponding control. Figure S2. No changed glucose tolerance and insulin sensitivity in miR-100 mice at start of the 16 weeks normal chow and high fat diet feeding period. (a–d) MiR-100 mice showed no altered glucose tolerance in the GTT (a, b) and insulin sensitivity in the ITT (c, d) compared to wildtype animals fed normal chow diet before the feeding period started (age 2–3 weeks, n = 5–6). (e–h) MiR-100 mice showed no altered glucose tolerance in the GTT (e, f) and insulin sensitivity in the ITT (g, h) compared to wildtype animals before the high fat feeding period started. (age 2–3 weeks, n = 13–14) Data represent mean values with SEM. n.s. = not significant, *P < 0.05, **P < 0.01 and ***P < 0.001 vs. the corresponding control. Figure S3. Reduced inflammation in the liver of miR-100 mice compared to wildtype after HFD feeding. (a, b) Quantitative real-time PCR analysis at the end of the HFD feeding period revealed a significant reduction of the inflammatory marker genes IL-1β and TNFα in miR-100 livers. (c) The expression of the oxidative stress marker Vnn1 is also significantly reduced in the liver of miR-100 overexpressing mice compared to wildtype. (age 18–19 weeks, n = 13–14) Data represent mean values with SEM. **P < 0.01 and ***P < 0.001 vs. the corresponding control. [file 10020_2021_364_MOESM2_ESM.docx]

**Figure S1**


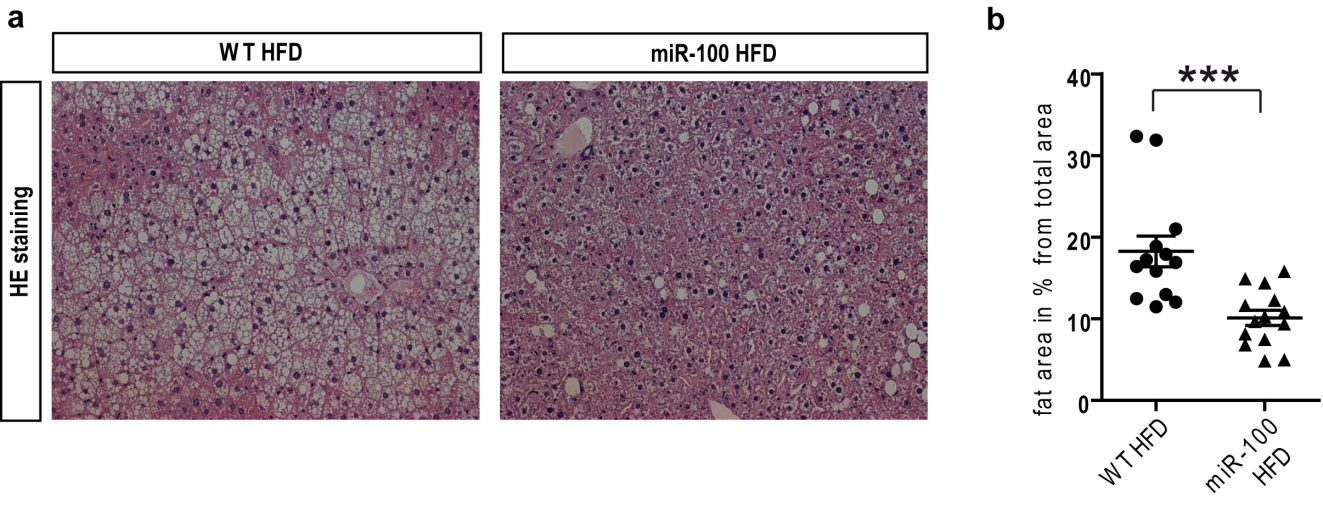


**Figure S1.** **Fat accumulation under high fat diet is reduced in miR-100 mice compared to wildtype.** (a) Representative pictures of paraffin preserved liver sections (8µm) stained for HE are shown. (b) The quantification of fat accumulation (white areas) was done using Image J software. (n=13-14) Data represent mean values with SEM. ***P<0.001 vs. the corresponding control.

**Figure S2**


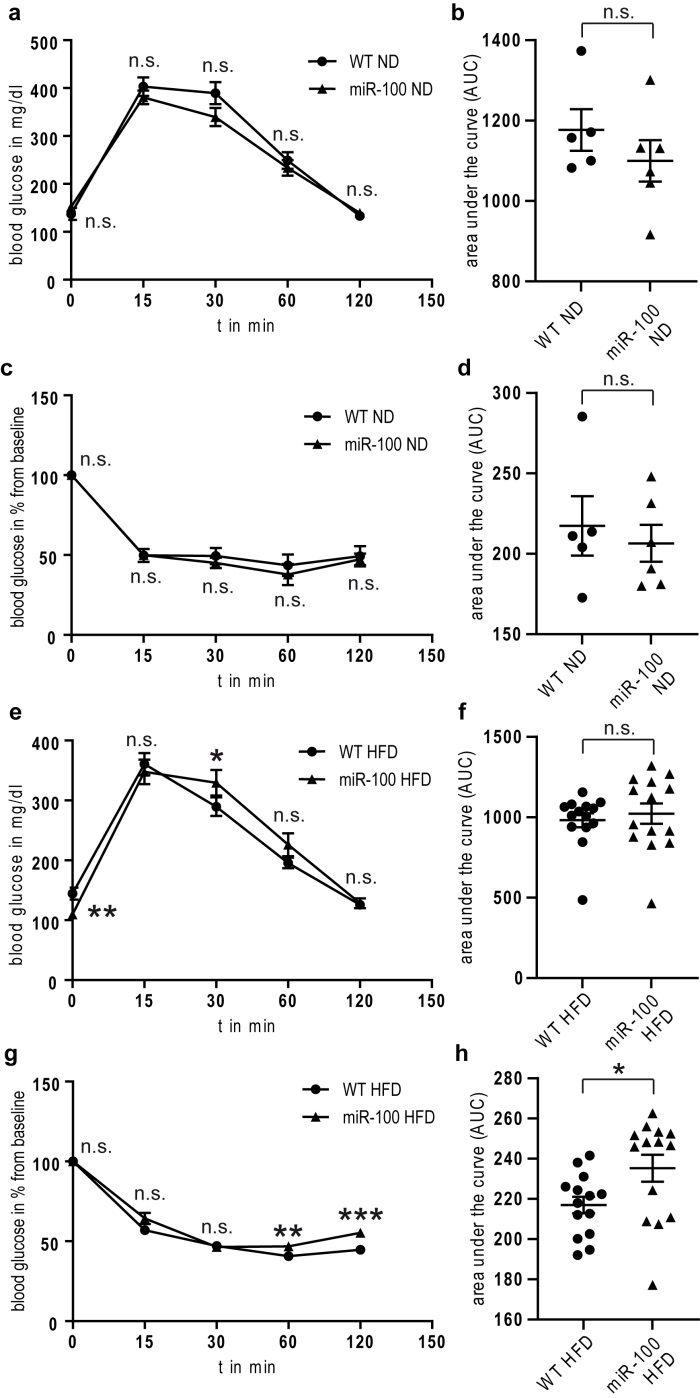


**Figure S2.** **No changed glucose tolerance and insulin sensitivity in miR-100 mice at start of the 16 weeks normal chow and high fat diet feeding period.** (a-d) MiR-100 mice showed no altered glucose tolerance in the GTT (a+b) and insulin sensitivity in the ITT (c+d) compared to wildtype animals fed normal chow diet before the feeding period started (age 2-3 weeks, n=5-6). (e-h) MiR-100 mice showed no altered glucose tolerance in the GTT (e+f) and insulin sensitivity in the ITT (g+h) compared to wildtype animals before the high fat feeding period started. (age 2-3 weeks, n=13-14) Data represent mean values with SEM. n.s.=not significant , *P<0.05, **P<0.01 and ***P<0.001 vs. the corresponding control.

**Figure S3**

**
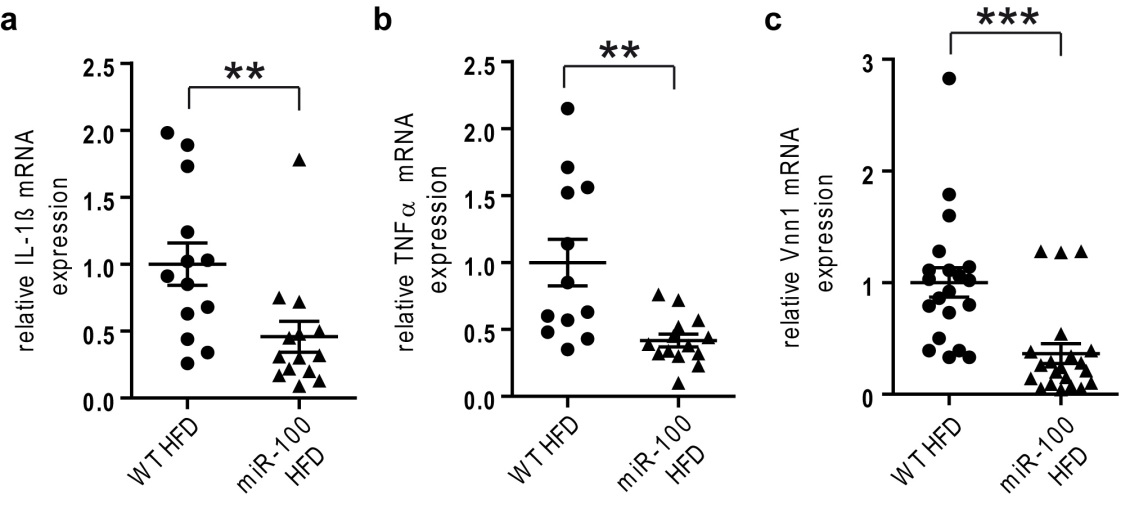
**

**Figure S3. Reduced inflammation in the liver of miR-100 mice compared to wildtype after HFD feeding.** (a+b) Quantitative real-time PCR analysis at the end of the high fat (HFD) feeding period revealed a significant reduction of the inflammatory marker genes IL-1β and TNFα in miR-100 livers. (c) The expression of the oxidative stress marker Vnn1 is also significantly reduced in the liver of miR-100 overexpressing mice compared to wildtype. (age 18-19 weeks, n=13-14) Data represent mean values with SEM. **P<0.01 and ***P<0.001 vs. the corresponding control.
